# Neonatal Skin Blistering and Denudement Caused by Epidermolysis Bullosa

**DOI:** 10.7759/cureus.113981

**Published:** 2026-08-05

**Authors:** Gavin Folkert, Anjali Aggarwal, Miltiadis Douvoyiannis

**Affiliations:** 1 School of Health Sciences, University of North Dakota, Grand Forks, USA; 2 Genetics, University of Minnesota Children's Hospital, Minneapolis, USA; 3 Pediatrics and Pediatric Infectious Diseases, Altru Health System, Grand Forks, USA; 4 Pediatrics, University of North Dakota, Grand Forks, USA

**Keywords:** epidermolysis bullosa, genetics, neonate, skin blister, skin denudement

## Abstract

The presented case of sharply localized neonatal blistering caused by autosomal dominant dystrophic epidermolysis bullosa due to a COL7A1 mutation illustrates the diagnostic challenges and clinical reasoning required in evaluating skin fragility disorders. It reinforces that epidermolysis bullosa should be considered early in the differential for congenital blistering, especially when distribution is localized to trauma-prone areas and infectious and autoimmune causes are excluded. Histopathology, immunofluorescence, and genetic testing remain essential for diagnosis. Supportive, multidisciplinary management and early counseling optimize outcomes, while advances in molecular therapy offer hope for transformative future care.

## Introduction

Epidermolysis bullosa (EB) encompasses a heterogeneous group of inherited mechanobullous disorders unified by skin fragility and blister formation following minimal trauma [1,2]. The prevalence of inherited EB was eight per one million live births in a national registry in the United States[3]. The disease arises from mutations in genes encoding proteins critical to the structural integrity of the dermal-epidermal junction, resulting in cleavage within or beneath the epidermis. Clinical severity varies widely, ranging from relatively mild, localized blistering in EB simplex to life-threatening forms of junctional or dystrophic EB associated with extracutaneous complications. The impact on patients and families can be lifelong and severely affect their quality of life. The neonatal period often represents the first point of recognition, and early diagnosis is critical for establishing prognosis, guiding care, and supporting families [1,2,4]. This report describes a neonate with sharply localized blistering confined to the feet and ankles, illustrating the diagnostic challenges of distinguishing EB from infectious or autoimmune causes and aims to increase the index of suspicion of the primary care providers about this rare but severe lifelong condition. 

## Case presentation

Presentation

A male neonate is born at 36 and 4/7 weeks, by cesarian section due to breech presentation to a G4P1021 mother. His birthweight is 2807 gr, appropriate for gestational age. Apgar scores were 8 at one and at five minutes. Pregnancy was complicated by maternal insulin-dependent gestational diabetes mellitus and severe pre-eclampsia. Sharply demarcated ulcerations and denuded skin localized to the bilateral feet and ankles are first noted shortly after the delivery. There is significant hesitation by the phlebotomists and the team to proceed with venipunctures and/or heel-sticks for monitoring the baby’s blood glucose. Given the notable skin lesions, he is admitted to the neonatal intensive care unit (NICU) for further management.

**Figure 1:**
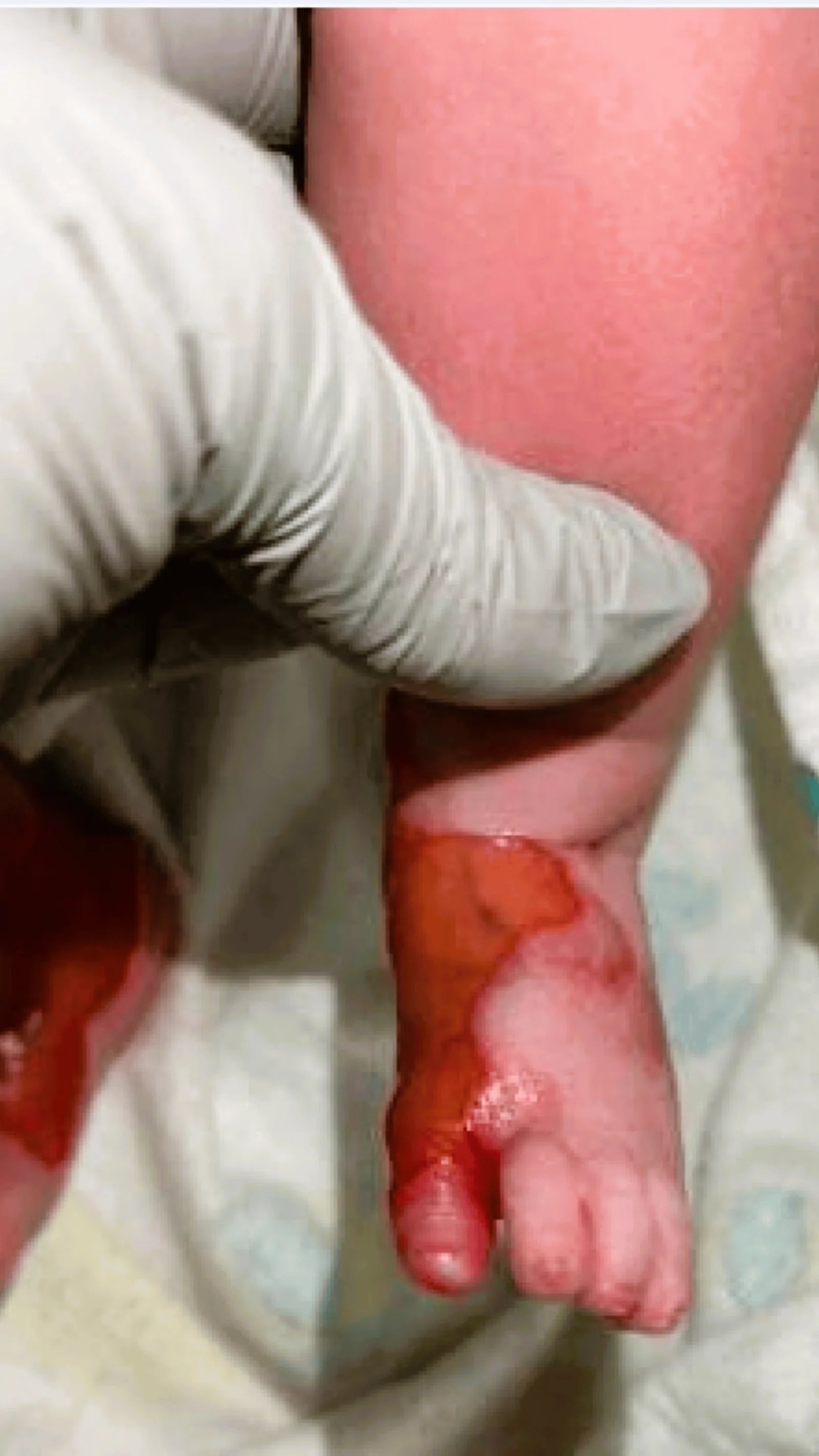
Frontal view of the left foot showing denuded skin over the dorsal surface.

**Figure 2:**
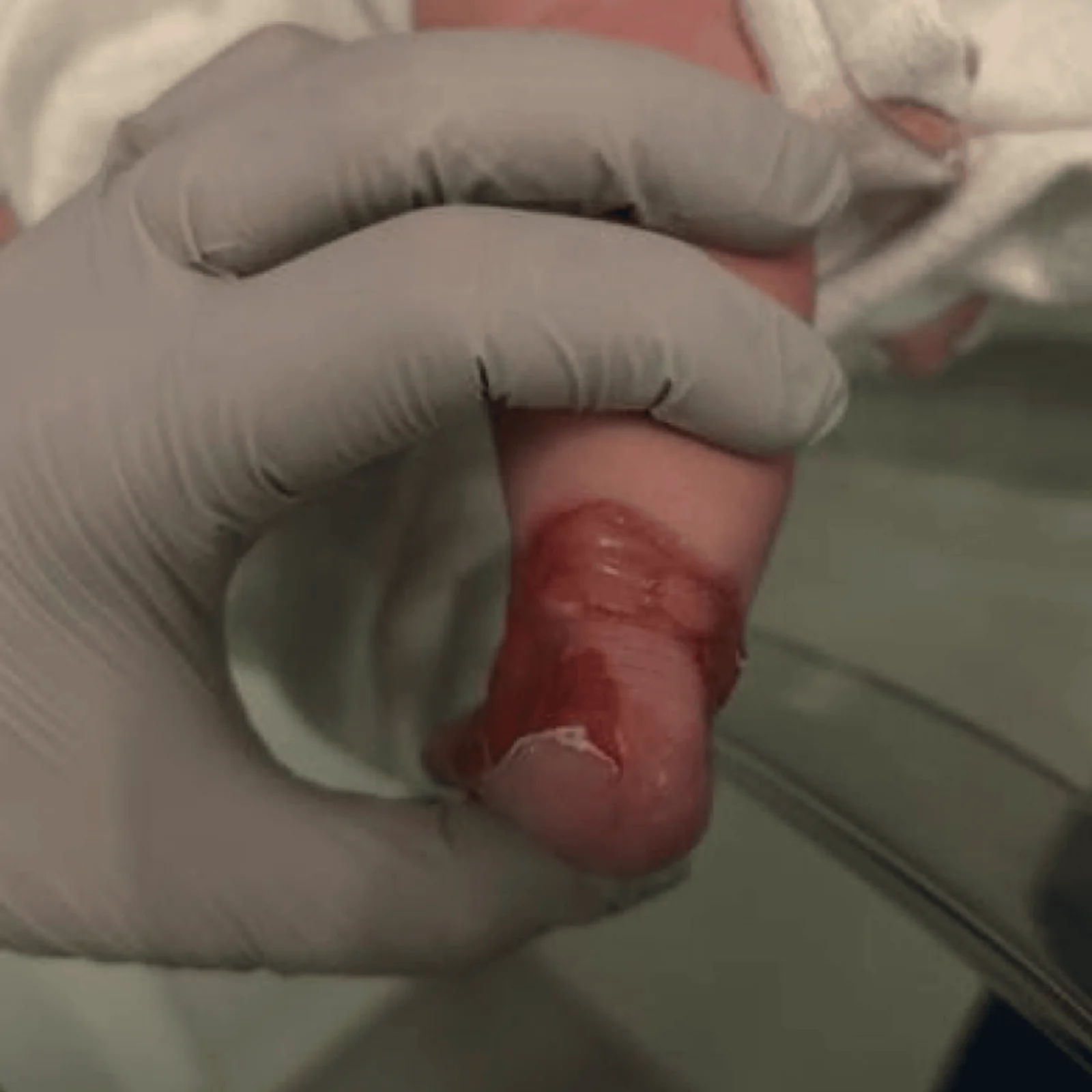
Posterior view of the right foot and ankle highlighting tense bullae and peeling skin.

**Figure 3:**
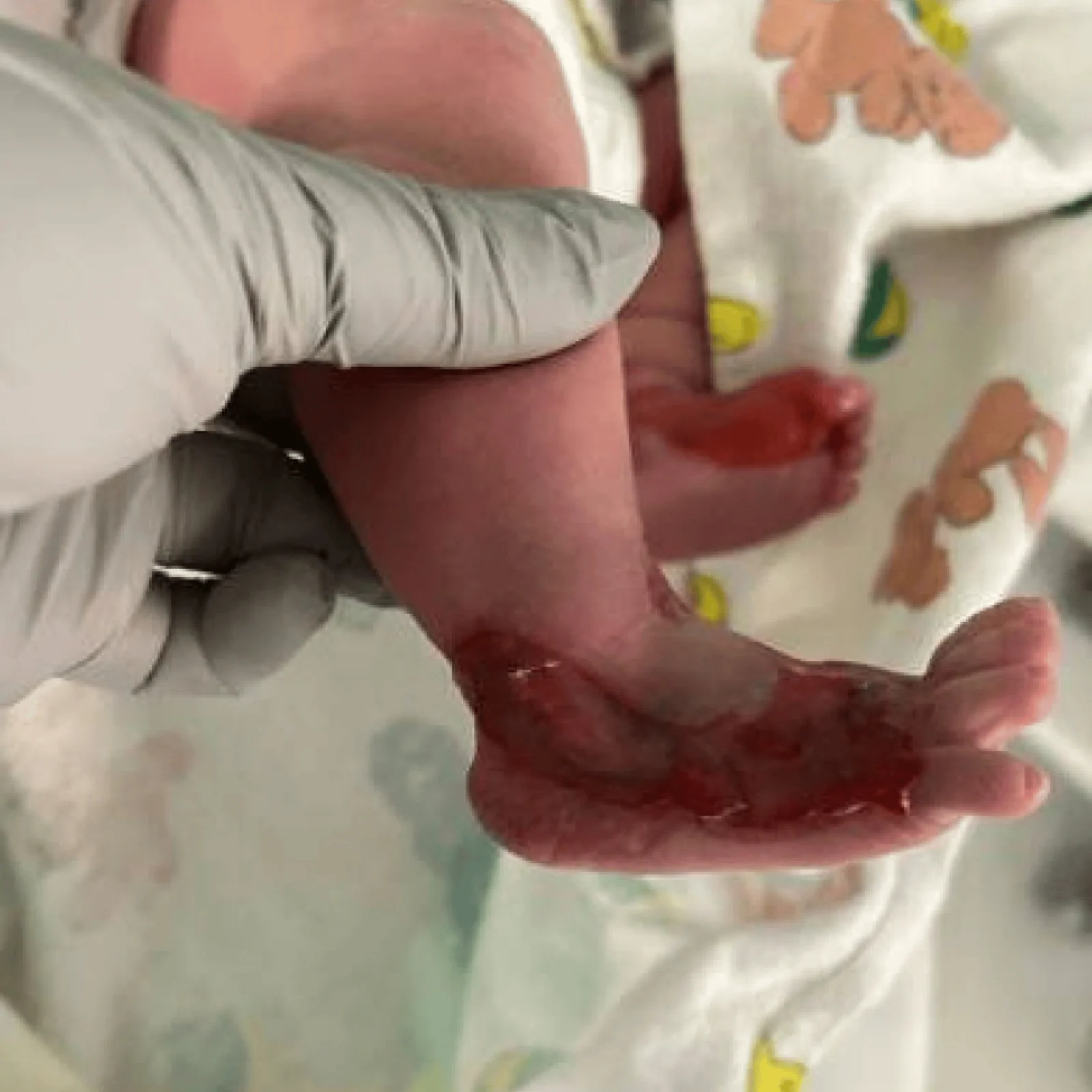
Lateral aspect of the right foot with denudement and bullae on lateral malleolus.

On examination, the baby is afebrile and hemodynamically stable. Denudement without surrounding erythema involves the plantar and dorsal surfaces of both feet and ankles. Blisters occur over the right heel. No lesions are present elsewhere, and mucous membranes are spared (Figures 1-3). Unlike infectious blistering, these lesions were confined to trauma-prone areas and lacked surrounding erythema. 

The remainder of the examination is unremarkable. Initial laboratory tests including a complete blood count and a complete metabolic panel are unremarkable, and blood culture remains negative. The differential diagnosis includes bullous impetigo or staphylococcal scalded skin syndrome (SSSS), neonatal autoimmune blistering disease (e.g., neonatal pemphigoid), congenital erosive and vesicular dermatosis and EB, among others. 

Diagnosis

Localized inherited EB in a neonate presenting with plantar and ankle ulcerations. Histopathology reveals subepidermal clefting without immuno-reactant deposition. Epidermolysis Bullosa Panel next generation sequencing panel was used to confirm the diagnosis and classify the subtype. A single pathogenic variant was identified COL7A1: NM_000094.4; c.6007G>A (p.Gly2003Arg), consistent with a diagnosis of autosomal dominant dystrophic EB.

Patient course

The neonate required NICU admission for further management. We were very careful during the short time of the management of the patient prior to transfer. So, Nikolsky sign was not actively assessed, no new lesions occurred, no significant need for use of pain medications occurred and the neonate was transferred after two days to a facility where genetic testing was available. Wound care with non-adherent dressings was initiated, and mupirocin ointment 2% was applied on the open areas. Bullae were lanced to avoid expansion. Daily baths with mild cleanser and foam dressings with Aquaphor were applied over the open areas as well as over the diaper area. Adhesive tapes were avoided and a softer tape with silicone was preferred. For discomfort, acetaminophen was used but in a restrictive way. Subsequent consultations with dermatology and pathology services were obtained. Biopsy and negative immunofluorescence suggested inherited EB. Following referral to a tertiary care university center, genetic testing confirmed the clinical diagnosis. A pathogenic missense variant in the COL7A1 gene was detected in this patient in the heterozygous state. This test result is consistent with a diagnosis of autosomal dominant EB due to a COL7A1 mutation. The infant was discharged in stable condition after six days with parental education. During this time, they also completed visits with dermatology and wound care.

## Discussion

Staphylococcus aureus-related bullous impetigo or SSSS are among the most common blistering conditions in neonates [5]. This infection typically presents with fragile bullae and superficial erosions, sometimes in widespread distribution, often accompanied by positive cultures or systemic illness. The absence of fever, negative cultures, and confinement of lesions to trauma-prone areas made infection less likely. Neonatal autoimmune blistering diseases, such as neonatal pemphigoid or pemphigus, may occur due to transplacental transfer of maternal antibodies. These cases often present with mucosal involvement or disseminated skin disease. Direct immunofluorescence is typically positive, distinguishing these conditions from EB. In our patient, negative immunofluorescence effectively excluded this category [6]. Congenital erosive and vesicular dermatosis, although rare, remains an important consideration. It is typically marked by generalized erosions and characteristic healing with reticulated scarring [7].

The histopathologic finding of subepidermal cleavage without immunoreactant deposition strongly supported inherited EB. Definitive diagnosis, however, rests on molecular analysis. Genetic testing not only clarifies the subtype but also informs prognosis, recurrence risk, and candidacy for emerging therapies. In this case, genetic confirmation provided the family with certainty, enabling focused counseling and care planning [1,2].

The diagnostic framework for EB relies on a stepwise approach: clinical assessment for patterns of blistering, distribution, mucosal involvement, and associated features; histopathology to determine the level of cleavage; immunofluorescence mapping or electron microscopy to identify disrupted proteins and exclude autoimmune disease; and genetic testing for definitive classification and precision counseling [1,2].

Management of EB in neonates is primarily supportive and requires a multidisciplinary team. Non-adherent dressings, avoidance of friction, meticulous wound care, and vigilant infection prevention are the cornerstones of therapy. Optimizing nutrition supports wound healing and growth, while dermatology and wound-care teams provide expertise in dressing selection and long-term skin care strategies [8]. Genetic counseling is indispensable, offering families clarity regarding inheritance patterns, recurrence risks, and available clinical trials.

Prognosis varies considerably. Localized EB simplex, often confined to the extremities, may improve as the child grows, sometimes with near resolution of blistering. By contrast, junctional EB and recessive dystrophic EB may carry significant morbidity, including chronic wounds, pseudosyndactyly, growth restriction, and risk of squamous cell carcinoma later in life [2,9]. Thus, early subtype determination is crucial. 

Emerging therapies are reshaping the EB landscape. Advances in molecular medicine have yielded promising interventions including gene therapy, protein replacement, and cell-based approaches. Early identification of EB subtypes enables families to access clinical trials and novel treatments as they become available. Although these therapies remain investigational, they highlight the importance of timely diagnosis and referral to EB specialty centers [9].

This case underscores several broader lessons. First, not all neonatal blistering is infectious. Second, localized blistering in trauma-prone sites should raise suspicion for EB, even in the absence of family history. Third, genetic testing is not merely confirmatory but essential to prognosis, management planning, and future therapeutic eligibility.

Finally, beyond clinical management, EB carries profound psychosocial implications. Families must navigate complex wound care routines, risk of recurrent infections, and uncertainty about long-term outcomes [10]. Early engagement with genetics, dermatology, and wound-care specialists provides not only medical guidance but also emotional support and connection to resources such as EB advocacy organizations. Seven simple and helpful tips for caring for people with EB can be found at NoBabyBlisters.org [11].

## Conclusions

This case highlights a localized, neonatal presentation of inherited EB, confined to the feet and ankles. The provider should consider inherited EB in neonates with congenital blistering localized to trauma-prone areas, especially in the absence of infection or autoimmunity. Histopathology and negative immunofluorescence help exclude inflammatory and autoimmune causes; genetic testing is required for definitive diagnosis and subtype differentiation. Management is supportive, involving gentle wound care, infection prevention, and multidisciplinary coordination. Advances in molecular medicine have yielded promising interventions including gene therapy, protein replacement, and cell-based approaches. Nevertheless the impact for the affected children and their families in some subtypes of EB is severe and lifelong. Early diagnosis enables accurate counseling, anticipatory guidance, and planning for long-term care.
